# Shenkang injection combined with alprostadil for chronic renal failure: A systematic review and meta-analysis

**DOI:** 10.3389/fmed.2023.982016

**Published:** 2023-04-06

**Authors:** Feng Xie, Tiantian Zhang, Pu Zhang, Xinliang Qu, Min Li, Wei Lan

**Affiliations:** ^1^College of Traditional Chinese Medicine, Xinjiang Medical University, Urumqi, Xinjiang Uygur Autonomous Region, China; ^2^Department of Pharmacy, Ankang Hospital of Traditional Chinese Medicine, Ankang, China

**Keywords:** Shenkang injection, alprostadil, chronic renal failure, meta analysis, Chinese medicine

## Abstract

**Objective:**

To systematically evaluate the clinical efficacy and safety of Shenkang injection (SKI) combined with alprostadil in the treatment of chronic renal failure (CRF).

**Method:**

Randomized controlled trials (RCTs) of Shenkang injection combined with alprostadil in CRF treatment were investigated by retrieving a total of 7 databases including CNKI, Wanfang database, VIP, CBM, PubMed, Embase and Cochrane Library, with the search time ranging from 2012 to now. Revman 5.2 software was used for data analysis, and Cochrane bias risk tool was used to evaluate the quality of the included literature. The final results were represented by relative risk (RR), mean difference (MD) and 95% confidence interval (95% CI).

**Results:**

A total of 20 RCTs and 1,573 patients were included in this study. Meta-analysis showed that the overall response rate (ORR) of the treatment group was superior to the control group [RR = 0.20, 95% CI (0.16, 0.25), *P* < 0.00001]. Compared with the control group, the treatment group achieved favorable improvement in terms of the creatinine clearance rate (Ccr) [MD = 9.48, 95% CI (8.73, 10.24), *P* < 0.00001], serum creatinine (Scr) [MD = −55.12, 95% CI (−63.42, −46.82), *P* < 0.00001], quantitative urine protein (Upro) [MD = −0.48, 95% CI (−0.53, −0.43), *P* < 0.00001], and blood urea nitrogen (BUN) [MD=-3.73, 95% CI (−4.08, −3.3) 7, *P* < 0.00001]. There was no statistical difference in the incidence of adverse reactions in each group.

**Conclusion:**

Currently, Shenkang injection combined with alprostadil has been widely used in clinical treatment of CRF due to the certain effect superior to other methods. However, its specific efficacy and safety need to be further verified through numerous large-scale clinical trials.

## 1. Introduction

The clinical-common chronic renal failure (CRF) can be developed from various diseases, such as primary glomerular disease, hypertensive nephropathy, diabetes nephropathy, drug-induced nephropathy, etc. (1–3). The annually growing prevalence of CRF mainly derives from the change of people's lifestyle, increasing number of diabetes and hypertension patients, as well as aging patients (4, 5). CRF is mainly manifested as disturbance of water and electricity medium and acid-base balance, abnormal endocrine function, retention of metabolites, etc. (6). The inevitable and reversible pathogenesis of CRF will eventually progress into uremia in the later stage, with dismal prognosis, which requires kidney transplantation or renal replacement therapy for life support, resulting in high expenditure and multiple complications. Currently, there is no specific drug to treat CRF (7).

Alprostadil is a kind of prostate drug aiming to expand renal vessels, significantly alleviating glomerular microcirculation, accelerating renal blood flow and increasing urine excretion, thus further promoting toxin excretion (8). The therapeutic efficacy of alprostadil is unsatisfactory despite its common application in CHF treatment. Recent years have witnessed certain outcomes in the application of integrated Chinese and Western medicine in clinical treatment of CRF (9–11). From the perspective of traditional Chinese medicine (TCM), CRF belongs to the categories of “edema,” “Stranguria syndrome,” and “kidney exhaustion.” CRF derives from its close association with the spleen and kidney, that is, the spleen and kidney deficiency, deficiency of both qi and yin, and the accumulation of dampness and turbidity. Shenkang injection (SKI), a kind of Chinese patent injection composed of rhubarb, salvia miltiorrhiza, astragalus and safflower, has been extensively used to treat CRF (12–14). On the one hand, pharmacological studies have found that SKI can decrease proteinuria by effectively alleviating anemia and renal microcirculation, thus inhibiting the pathological proliferation of kidneys, which is conducive to reducing the damage to the kidneys, promoting the repair of damaged tissues, eventually improving renal function (15). On the other hand, SKI aims to reduce the infiltration of inflammatory cells by alleviating glomerulosclerosis and interstitial fibrosis, thus reducing the level of serum creatinine and blood urea nitrogen (16).

The combination of SKI and alprostadil has been shown to be synergistic, with demonstrable therapeutic effect (17–19). Considering that a single study may not represent the overall effect, comprehensive collection and reference were conducted on data from CRF treatment to objectively evaluate the efficacy of Shenkang injection combined with alprostadil, so as to determine their efficacy and safety, and provide evidence for the evidence-based medicine system.

## 2. Materials and methods

### 2.1. Inclusion criteria and exclusion criteria

#### 2.1.1. Research type

Clinical randomized controlled trials (RCTS) of Shenkang injection combined with alprostadil in CRF treatment were included in this study.

#### 2.1.2. Research object

Patients with obvious clinical symptoms such as metabolic disorder, metabolite retention, chronic kidney disease or all diseases involving the kidney, the endogenous creatinine clearance rate (Ccr) < 80 mL/min, and the serum creatinine (Scr) >133 μmol/L according to the diagnostic criteria of the Guidelines for the Diagnosis and Treatment of Chronic Renal Failure (37). Patients diagnosed with deficiency of both qi and yin, presenting with at least 4 symptoms including nausea and vomiting, pale face, bloating, qi deficiency, anorexia, night sweats, bitter mouth, dry tongue, etc. according to the Guidelines for the Diagnosis and Treatment of Chronic Renal Failure by Integrated Traditional Chinese and Western Medicine (38).

#### 2.1.3. Interventions

The control group was administrated with alprostadil, Shenkang injection or routine treatment, while the treatment group was administrated with Shenkang injection combined with alprostadil.

#### 2.1.4. Outcome indicators

The outcome indicators were clinical efficacy, serum creatinine (Scr), blood urea nitrogen (BUN), urinary protein quantification (Upro), creatinine clearance rate (Ccr), aromatase (ArE) and adverse reaction rate.

#### 2.1.5. Exclusion criteria

Non-RCT research, repeated published research, related literature review, research, research with incomplete clinical data and inconsistent outcome indicators, patients undergoing renal replacement therapy such as dialysis.

### 2.2. Search strategy

A total of 7 databases including CNKI, Wanfang database, VIP, CBM, PubMed, Embase and Cochrane Library were retrieved, with the search time ranging from 2012 to now. The retrieval followed the PICOS principle, that is, P: CRF patients; I: Shenkang injection+alprostadil; C: Alprostadil, Shenkang injection or routine treatment; O: Relevant CRF indexes; S: Randomized controlled trial. We adopted the search strategy of combining Chinese and English keywords. key words: Shenkang injection, alprostadil, chronic renal failure, chronic renal insufficiency, chronic kidney disease.

### 2.3. Data extraction and quality evaluation of document methodology

According to the previously set inclusion and exclusion criteria, the two researchers used Note Express document management software to independently screen the literature. Any difference was resolved by the third researcher through negotiation. After screening the qualified literature and extracting relevant data, the two researchers used the bias risk assessment tool recommended by Cochrane System Reviewer Manual to evaluate the bias risk of the included RCTs. The evaluation methods included random scheme, concealment of distribution scheme, blind method, integrity of result data, selective report and other biased sources The evaluation of each study was divided into low risk, unclear risk and high risk.

### 2.4. Evidence quality evaluation

The GRADE system was used to evaluate the quality of evidence from the included literature. The limitations, inconsistency, indirection, inaccuracy and publication bias of the outcome indicators were, respectively evaluated, thus accordingly dividing the quality of evidence (39). Thereinto, the quality of RCT evidence was initially preset as “high.” Of all 5 degradation factors, only one was degraded to “medium,” two to “low,” and three to “extremely low.” The difference between the true value and the estimated value increased from “high level” to “extremely low level.” Methodological quality evaluation and evidence quality grading were independently conducted by two researchers and cross-checked. Any problem was solved by both parties through negotiation.

### 2.5. Statistical methods

RevMan 5.2 software provided by Cochrane was used for meta-analysis. In outcome indicators, the clinical efficacy and adverse reactions were classified as binary variables. The risk ratio (RR) was used for analysis and statistics, and the combined effect amount of mean difference (MD) was used for continuous variables. Each effect amount was represented by 95% confidence interval (95% confidence interval, CI). *P*-value and I^2^ were used to evaluate the heterogeneity among the groups, *P* < 0.05 was considered to be statistically significant. The fixed effect model was used for analysis under the condition of small data heterogeneity between groups (*P* > 0.10 or *I*^2^ ≤ 50%), otherwise the combined effect amount was calculated using the random effect model under the condition of significant data heterogeneity (*P* ≤ 0.10 or *I*^2^ ≥ 50%). Significant heterogeneity was eliminated by subgroup analysis or sensitivity analysis.

## 3. Results of literature search

**Figure 1:**
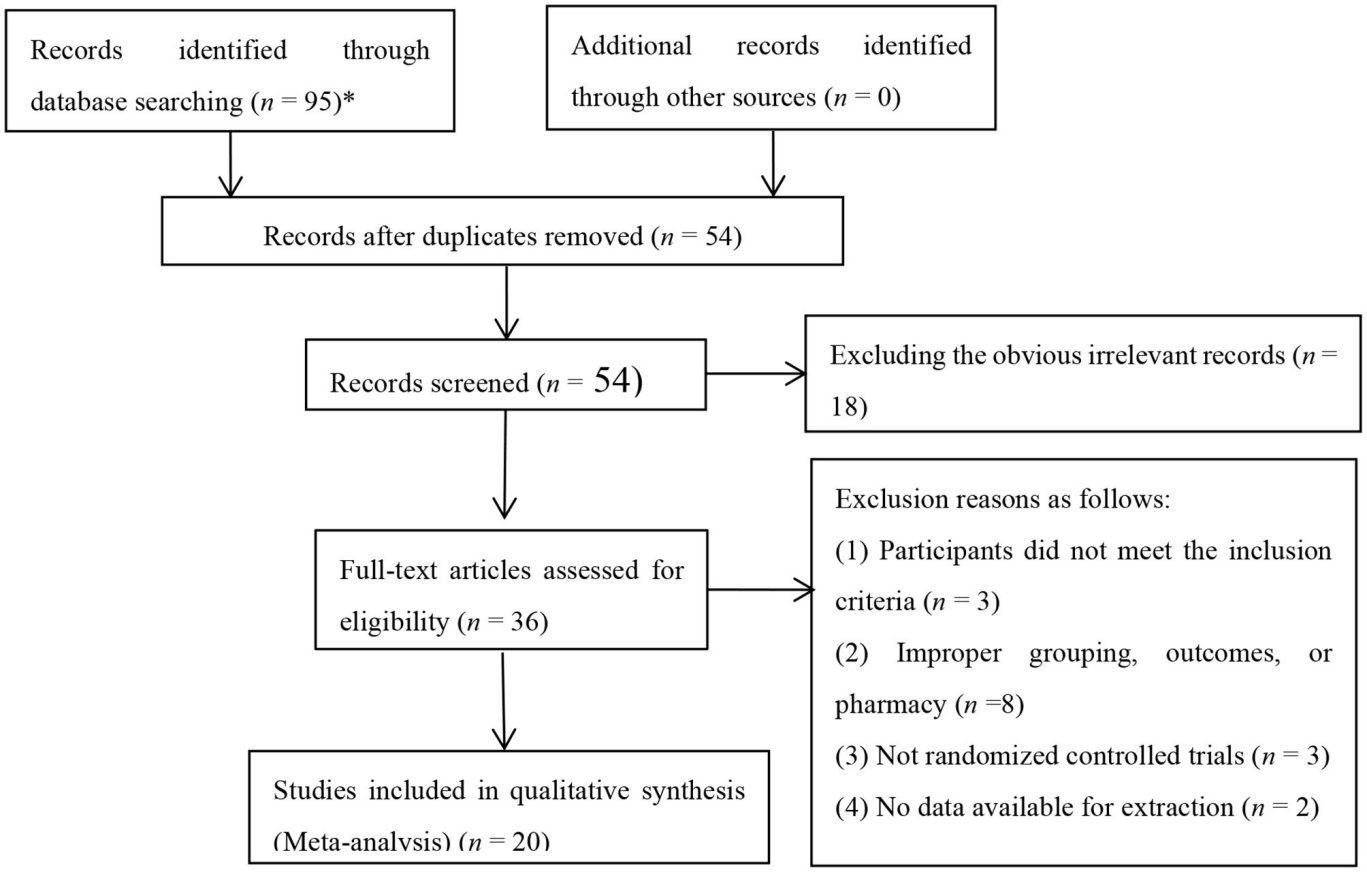
Flow chart of study selection process.

Ninety-five relevant documents were initially retrieved. After eliminating duplicate documents using Note Express, a total of 54 papers were obtained. Non-randomized controlled trials were eliminated after reading the title and abstract, and further the full text. Finally 20 RCTs were included after comparing the inclusion and exclusion criteria, with the published time from 2012 to 2020 (Figure 1).

### 3.1. Baseline of the included studies

A total of 1,573 patients in 20 papers were included, including 789 cases in the treatment group and 784 cases in the control group, with the shortest course of 14 days and the longest course of 28 days. In terms of treatment measures in the control group, alprostadil accounted for 11 items, Shenkang injection accounted for 5 items, and routine treatment accounted for 4 items. Observation indicators included clinical efficacy, Scr, BUN, UPro, Ccr, ArE, and adverse reactions (Table 1).

**Table 1:** Principal characteristics of the studies included in the meta-analysis.

**No**.	**Included study**	**Sample size (T/C)**	**Experimental group**		**Control group**		**course of treatment**	**Evaluation indicators**
			**Treatment**	**Age (years)**	**Treatment**	**Age (years)**		
1	Chen et al. (17)	45/45	Alprostadil+Shenkang Injection	48.1 ± 2.7	Shenkang Injection	47.5 ± 2.3	14 d	(1)(2)(3)(4)
2	Ma et al. (18)	31/31	Alprostadil+Shenkang Injection	67.5 ± 4.2	Alprostadil	67.5 ± 4.2	14 d	(1)(2)(3)(4)
3	Wang et al. (19)	43/43	Alprostadil+Shenkang Injection	42.6 ± 9	Conventional treatment	43.6 ± 9	14 d	(2)(3)(4)(6)(7)(8)
4	Zhang et al. (20)	46/46	Alprostadil+Shenkang Injection	52.45 ± 8.46	Conventional treatment	52.37 ± 8.24	28 d	(1)(2)(3)(4)(5)
5	Zhang et al. (21)	30/30	Alprostadil+Shenkang Injection	65.08 ± 3.46	Conventional treatment	65.06 ± 3.36	28 d	(1)(2)(3)(4)(8)
6	Wang et al. (22)	40/40	Alprostadil+Shenkang Injection	65.8 ± 8.8	Alprostadil	67.1 ± 9.1	28 d	(2)(3)(4)(5)(9)
7	Chen et al. (23)	41/41	Alprostadil+Shenkang Injection	73.4 ± 1.5	Shenkang Injection	74.0 ± 1.6	28 d	(1)(2)(3)(4)(5)
8	Feng et al. (24)	41/41	Alprostadil+Shenkang Injection	49.0 ± 8.4	Alprostadil	53.5 ± 9.7	28 d	(1)(2)(3)(4)(5)
9	Zou et al. (25)	35/35	Alprostadil+Shenkang Injection	57.0 ± 10.3	Alprostadil	56.5 ± 10.8	28 d	(1)(2)(3)(4)(5)
10	Zhao et al. (26)	33/33	Alprostadil+Shenkang Injection	68.55 ± 6.36	Shenkang Injection	68.63 ± 6.75	28 d	(1)(2)(3)(4)
11	Lan et al. (27)	41/41	Alprostadil+Shenkang Injection	65.7 ± 3.7	Shenkang Injection	65.3 ± 3.8	28 d	(2)(3)(4)
12	Liu et al. (28)	46/46	Alprostadil+Shenkang Injection	38.25 ± 7.63	Alprostadil	39.05 ± 7.99	28 d	(1)(2)(4)(5)
13	Li et al. (29)	25/25	Alprostadil+Shenkang Injection	39.64 ± 7.23	Alprostadil	37.56 ± 4.82	14 d	(1)(2)(3)(4)
14	Gao et al. (30)	48/48	Alprostadil+Shenkang Injection	57.18 ± 11.96	Alprostadil	56.34 ± 10.87	28 d	(1)(2)(3)(4)(5)
15	Dai et al. (31)	42/41	Alprostadil+Shenkang Injection	57.34 ± 7.45	Alprostadil	57.16 ± 7.52	28 d	(1)(2)(3)(4)
16	Liu et al. (32)	30/30	Alprostadil+Shenkang Injection	76.20 ± 5.04	Shenkang Injection	76.43 ± 4.24	28 d	(1)(2)(3)(4)
17	Feng et al. (33)	54/53	Alprostadil+Shenkang Injection	44.1 ± 5.0	Alprostadil	44.9 ± 5.3	14 d	(2)(3)(4)(5)(6)(7)
18	Zhang et al. (34)	54/54	Alprostadil+Shenkang Injection	56.12 ± 10.43	Alprostadil	57.39 ± 11.78	28 d	(1)(2)(3)(4)
19	Zhang et al. (35)	44/43	Alprostadil+Shenkang Injection	48.0 ± 3.0	Alprostadil	48.0 ± 3.0	28 d	(1)(2)(3)(4)(7)(8)
20	Xiao et al. (36)	47/45	Alprostadil+Shenkang Injection	43.7 ± 9.0-	Conventional treatment	43.7 ± 9.0	14 d	(2)(3)(4)

(1) Total effective rate; (2) Scr; (3) BUN; (4) Upro; (5) Ccr; (6) TG; (7) TC; (8) ArE; (9) Adverse reactions.

### 3.2. Risk of bias assessment of the included studies

**Figure 2:**
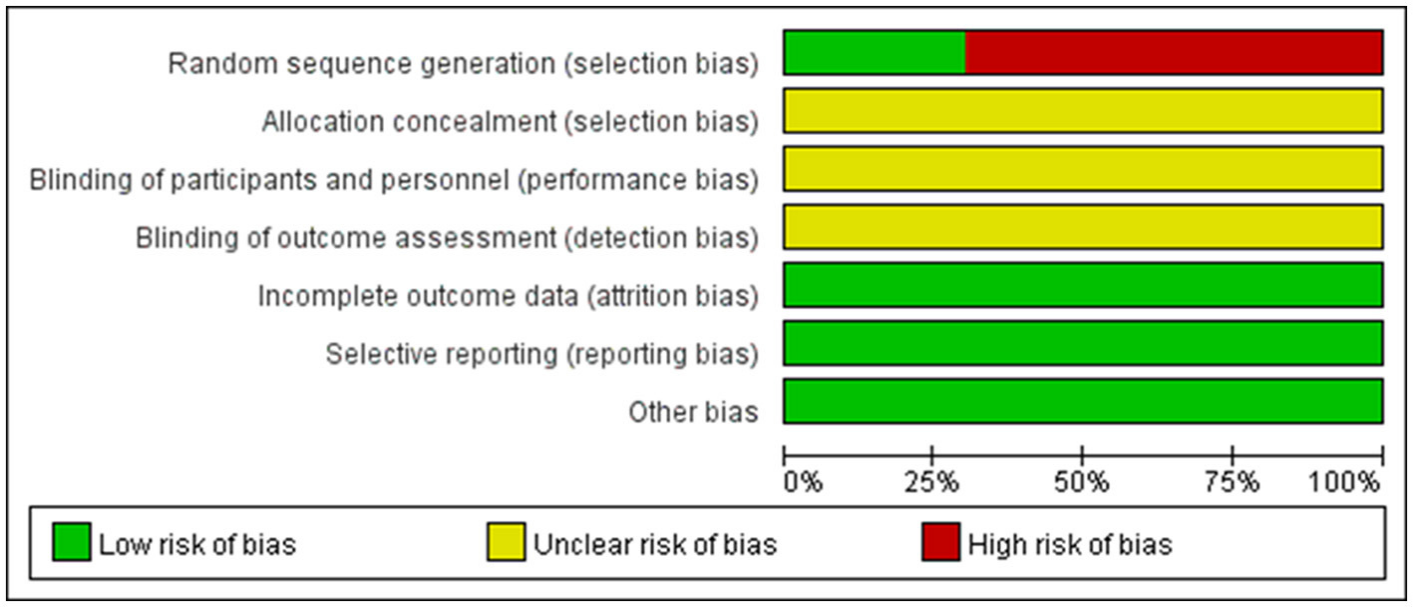
Risk of bias graph and summary. Red, high risk; green, low risk; yellow, unclear risk.

According to the risk of bias assessment method recommended by Cochrane, all studies were randomized trials. In the included 20 articles, 6 of them described specific randomization methods, and the remaining 14 did not specify random allocation methods. None of the research results lacked outcome data, and they were all not selectively reported. However, there was no detailed description of blind method and allocation concealment (Figure 2).

### 3.3. Results of meta-analysis

#### 3.3.1. Clinical efficacy

**Figure 3:**
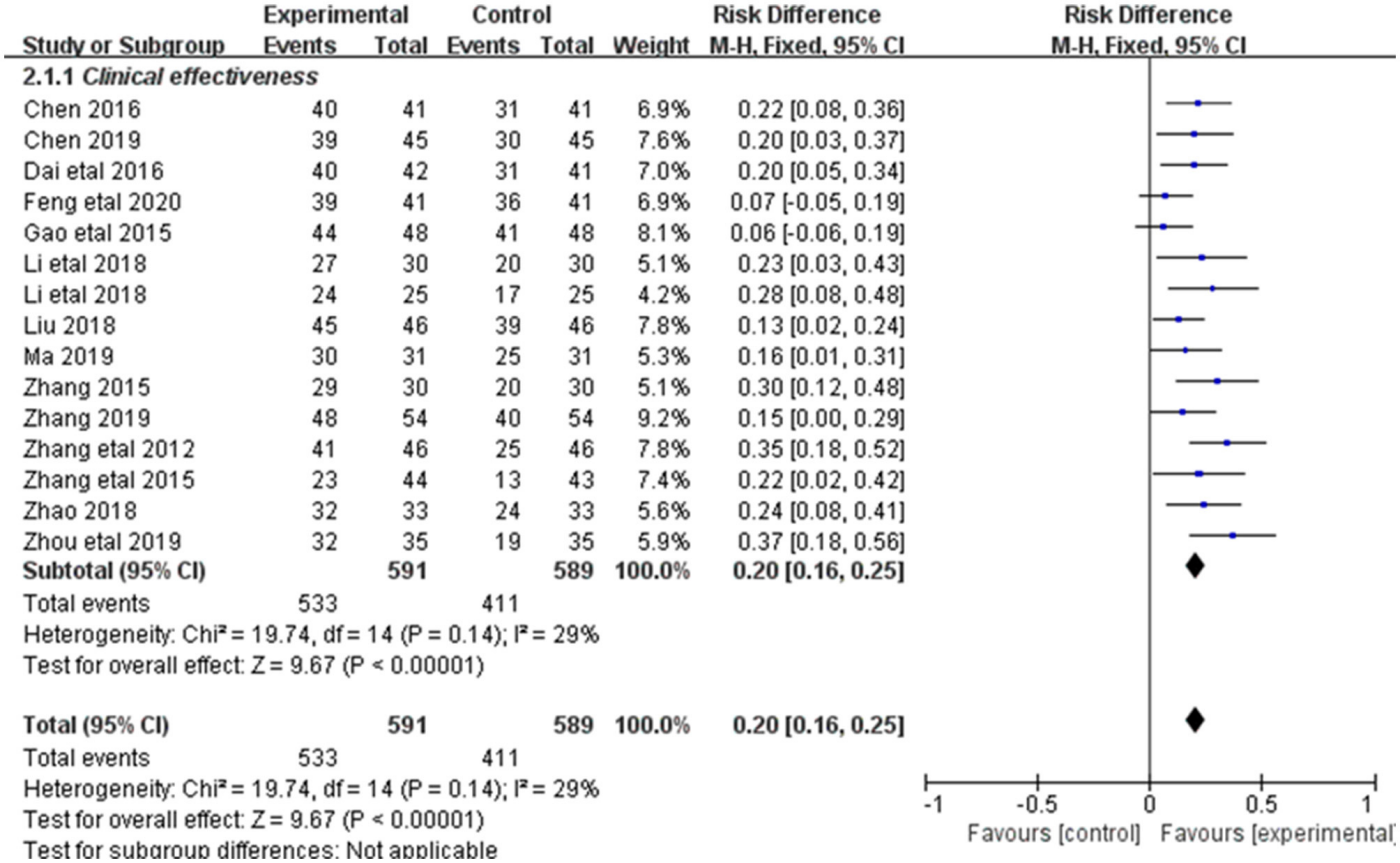
Forest plot of the total effectiveness rate.

Changes in clinical efficacy were reported in 15 of the 20 included studies (Figure 3). Results showed no heterogeneity among the studies (DF=14, *P* = 0.14, *I*^2^ = 29%). Therefore, the fixed effect model was used for analysis. The overall response rate (ORR) of the combined treatment group was significantly higher than that of the control group [RR = 0.2, 95% CI = (0.16, 0.25)], with statistical significance (*P* < 0.00001).

#### 3.3.2. SCR

**Figure 4:**
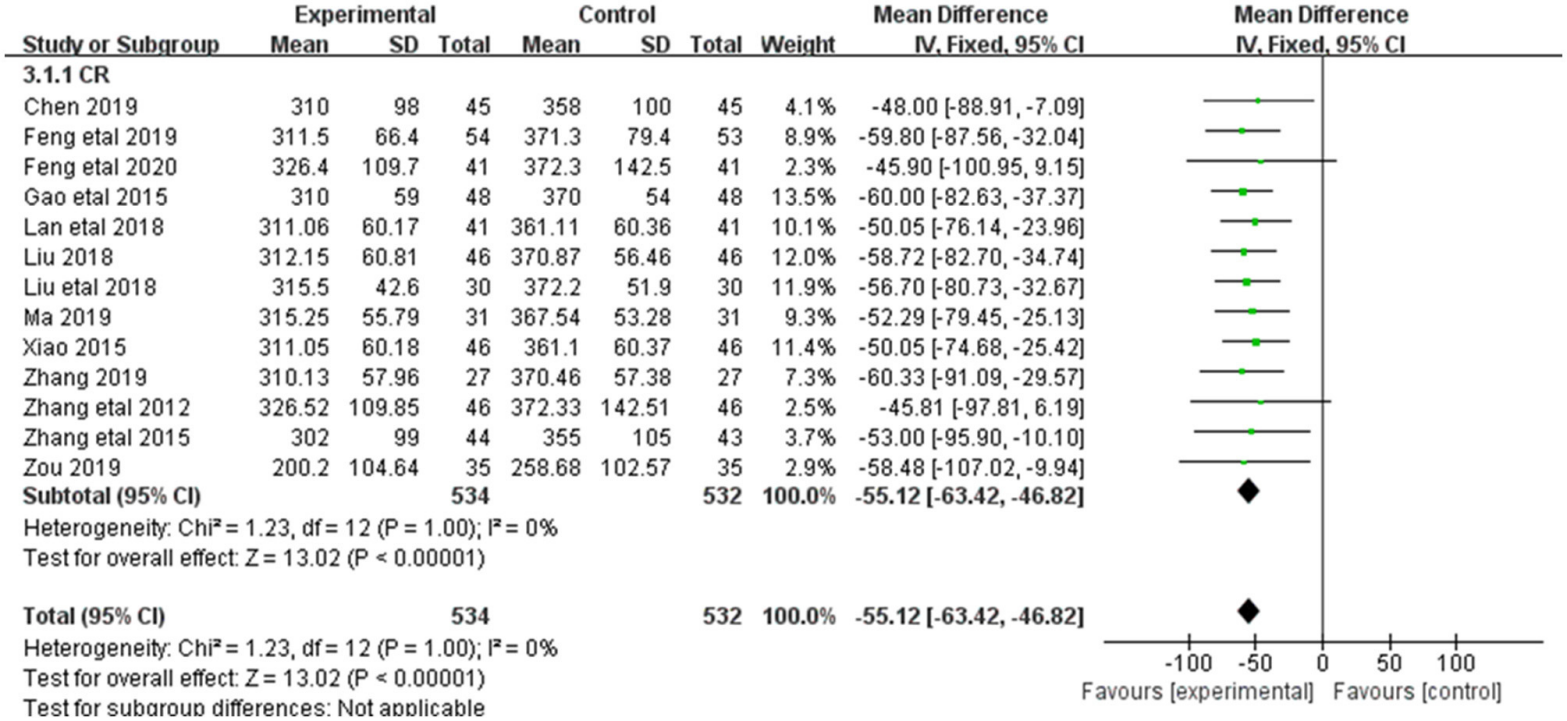
Forest plot of analysis of the Scr of treating CHF.

Alleviation in SCR was reported in 13 of the 20 included studies (Figure 4). The research results showed no heterogeneity among the studies (DF = 12, *P* = 1, *I*^2^ = 0%). Therefore, the fixed effect model was used for analysis. The effect of Shenkang injection combined with alprostadil in treating CRF was superior to the control group [MD = 9.48, 95% CI (8.73, 10.24)], with statistical significance (*P* < 0.00001).

#### 3.3.3. BUN

**Figure 5:**
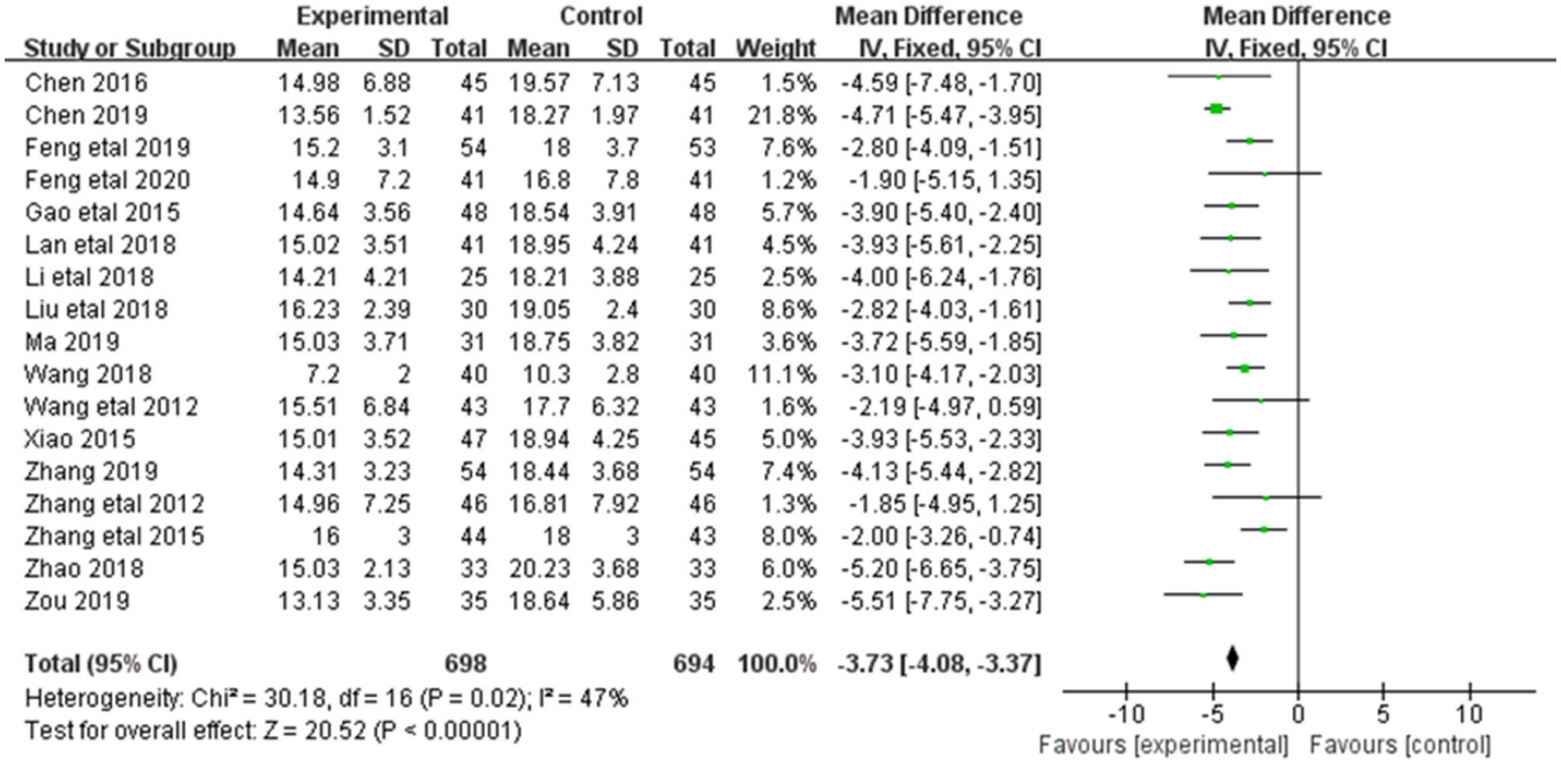
Forest plot of analysis of the BUN of treating CHF.

Alleviation in BUN was reported in 17 of the 20 included studies (Figure 5). The research results showed no heterogeneity among the studies (DF = 16, *P* = 0.02, *I*^2^ = 47%). Therefore, the fixed effect model was used for analysis. The alleviation of BUN in the combined treatment group was superior to the control group [MD = −3.73, 95%CI = (−4.08, −3.37)], with statistical significance (*P* < 0.00001).

#### 3.3.4. Upro

**Figure 6:**
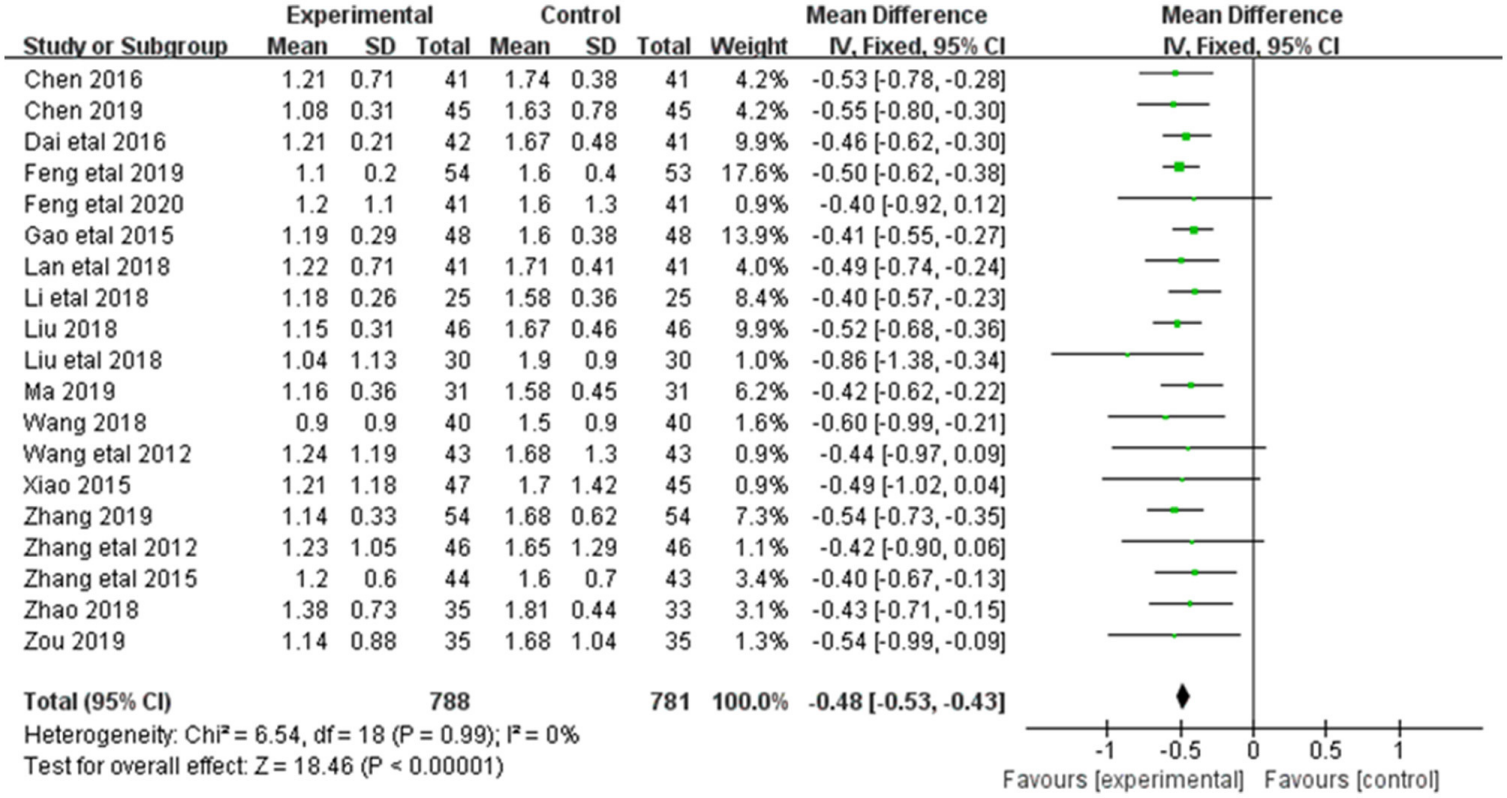
Forest plot of analysis of the Upro of CHF.

Alleviation in Upro was reported in 19 of the 20 included studies (Figure 6). The research results showed no heterogeneity among the studies (DF = 18, *P* = 0.99, *I*^2^ = 47%). Therefore, the fixed effect model was used for analysis. The alleviation of Upro in the combined treatment group was superior to the control group [MD = −0.48, 95%CI = (−0.53, −0.43)], with statistical significance (*P* < 0.00001).

### 3.3.5. Ccr

**Figure 7:**
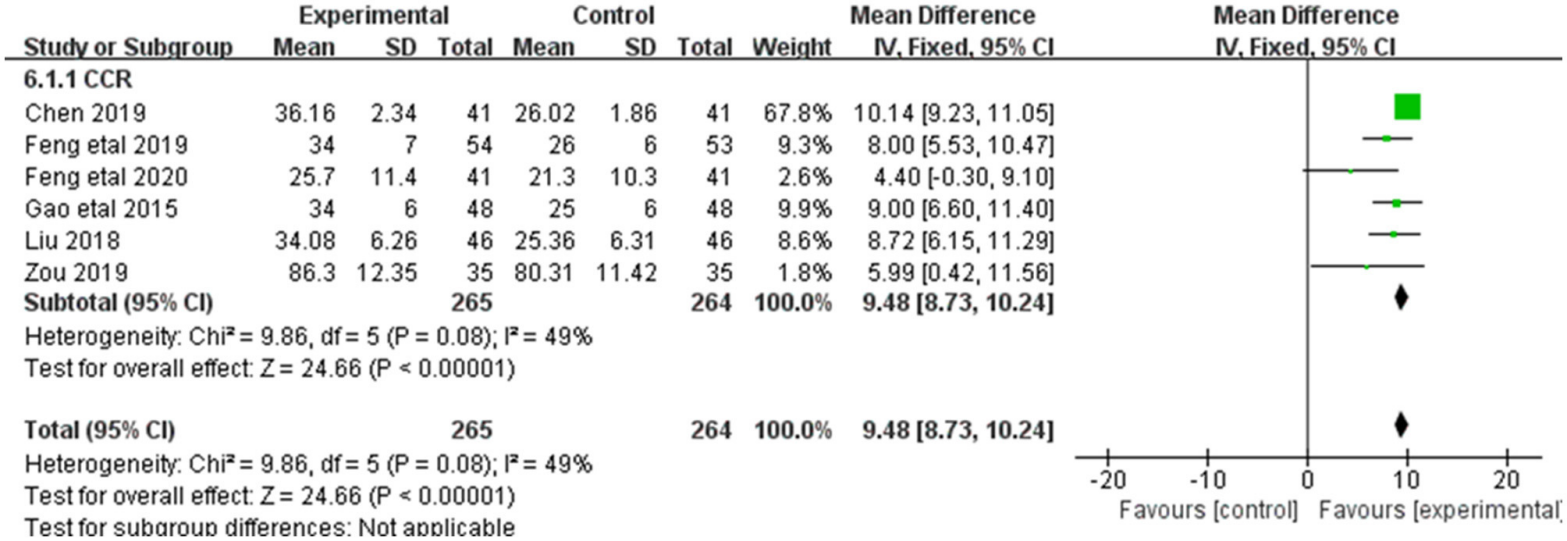
Forest plot of analysis of the Ccr of CHF.

Alleviation in Ccr was reported in 6 of the 20 included studies (Figure 7). The research results showed no heterogeneity among the studies (DF = 5, *P* = 0.08, *I*^2^ = 49%). Therefore, the fixed effect model was used for analysis. The alleviation of Ccr in the combined treatment group was superior to the control group [MD = 9.48, 95%CI = (8.37, 10.24)], with statistical significance (*P* < 0.00001).

#### 3.3.6. ArE

**Figure 8:**
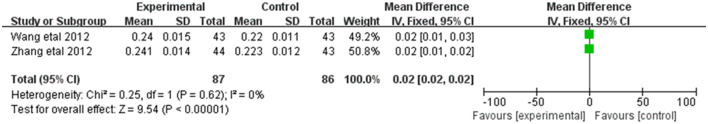
Forest plot of analysis of the ArE of treating CHF.

Alleviation in ArE was reported in 2 of the 20 included studies (Figure 8). The research results showed no heterogeneity among the studies (DF = 1, *P* = 0.62, *I*^2^ = 0%). Therefore, the fixed effect model was used for analysis. The alleviation of ArE in the combined treatment group was superior to the control group [MD = 0.02, 95%CI = (0.02, 0.02)], with statistical significance (*P* < 0.00001).

#### 3.3.7. Adverse reactions

**Figure 9:**
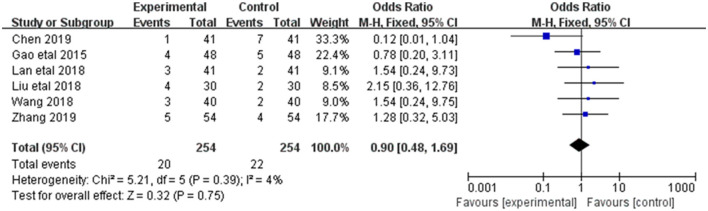
Forest plot of analysis of the adverse reactions of treating CHF.

Adverse reactions were reported in 6 of the 20 included studies (Figure 9). The research results showed small heterogeneity among the studies (DF = 5, *P* = 0.30, *I*^2^ = 4%). Therefore, the fixed effect model was used for analysis. Meta-analysis showed no statistical significance between the treatment group and the control group [MD = 0.90, 95%CI = (0.48,1.69), *P* = 0.75].

#### 3.3.8. Assessment of publication bias

**Figure 10:**
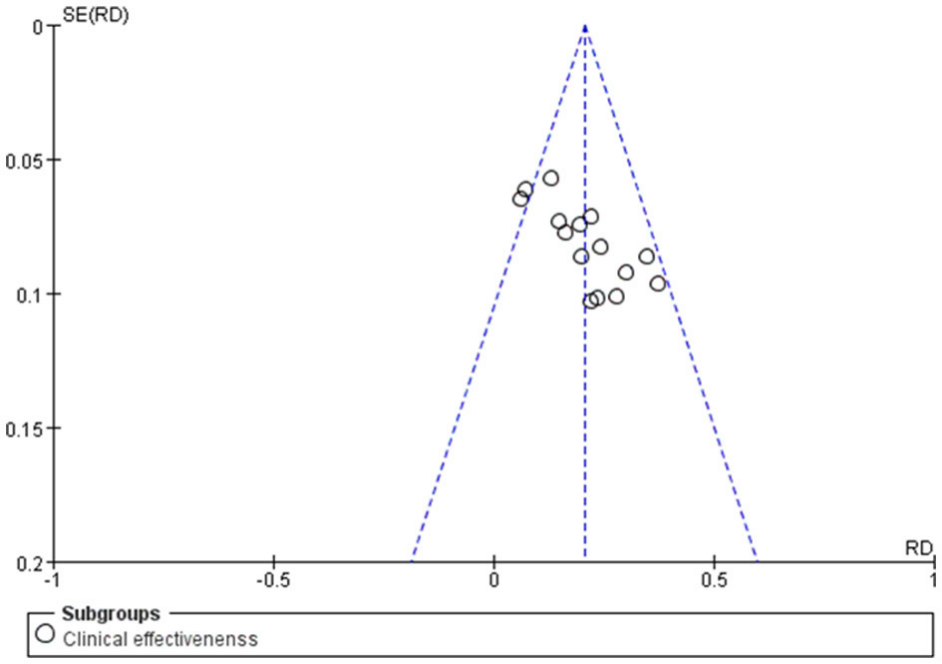
Funnel plot of the total efficacy of Shenkang injection combined with alprostadil and control drugs.

**Figure 11:**
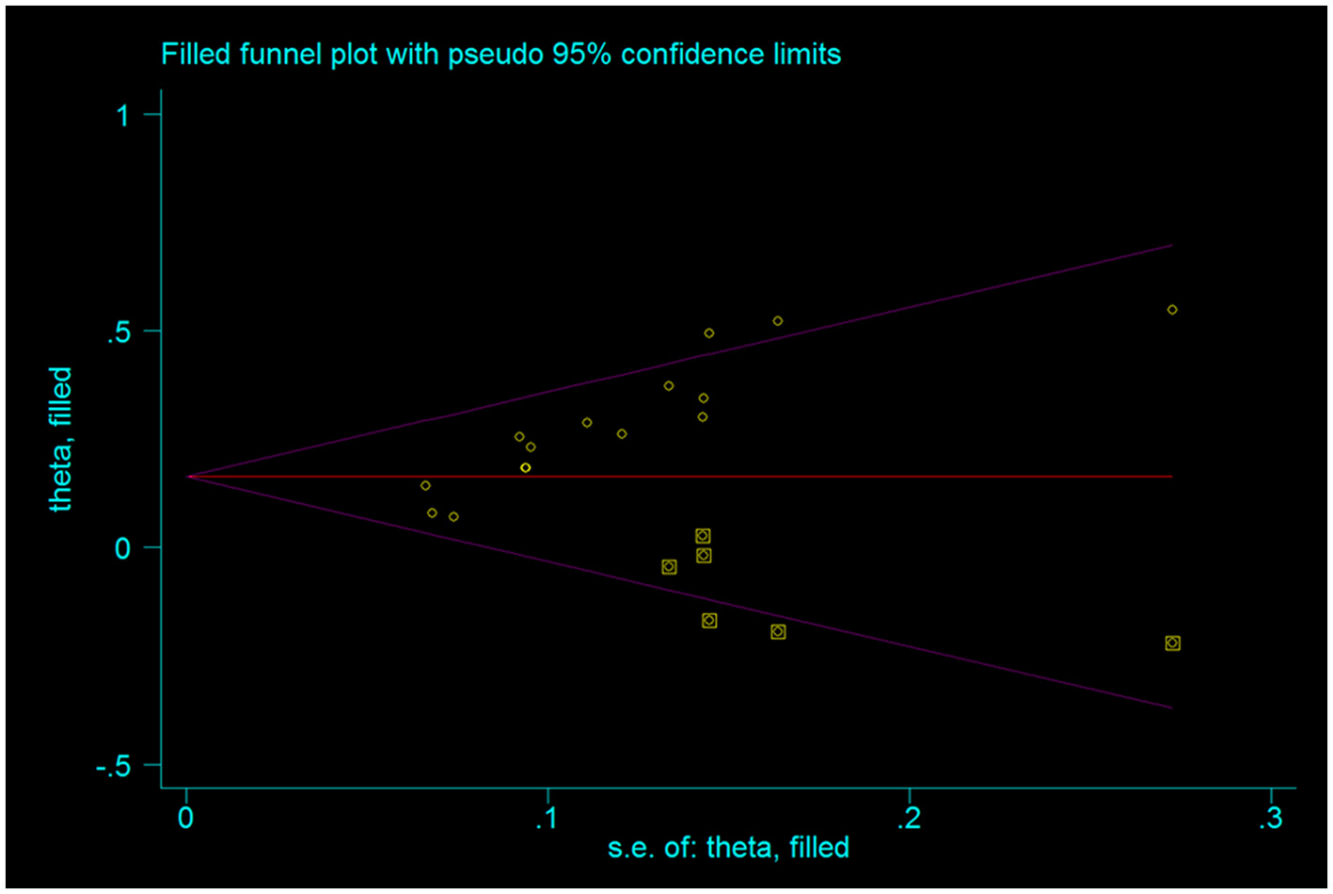
Funnel chart after trimming.

The publication bias test was conducted for the outcome indicators of more than 10 studies in the report (Figure 10). The relatively asymmetric funnel graph of total effective outcome indicators indicated the existence of certain publication bias. Egger method test showed that the index had a certain publication bias (*P* = 0.000 < 0.05), and cutting compensation method was used for analysis. The estimated RR point and 95% CI before and after pruning were 1.29 [1.22, 1.37] and 1.177 [1.123, 1.234], respectively. The adjusted statistics and significance were Z = 6.806, *P* < 0.05, and the adjusted conclusion was not reversed, indicating the relative stability of the conclusion (Figure 11).

#### 3.3.9. Sensitivity analysis

Using successively eliminating each study, sensitivity analysis was conducted on the remaining results. The Meta-analysis results showed no significant change in each effect value after excluding any study, indicating the stability and reliability of the results.

### 3.4. Results of GRADE evidence quality evaluation

The GRADE system was used to classify the quality of the main outcome indicators of 20 documents (Table 2). All outcome indicators were of medium and low quality, without high-quality evidence, suggesting that the results need to be further confirmed by a large-scale, multi-center RCT.

**Table 2:** Outcome index GRADE evidence quality evaluation.

**Shenkang injection combined with alprostadil compared to alprostadil for chronic renal failure**						
**Patient or population: patients with [health problem] Settings: Intervention: Clinical effectiveness**						
**Outcomes**	**Illustrative comparative risks** ^*^ **(95% CI)**		**Relative effect (95% CI)**	**No of Participants (studies)**	**Quality of the evidence (GRADE)**	**Comments**
	**Assumed risk**	**Corresponding risk**				
	**Control**	**Clinical effectiveness**				
Total effective rate	**Study population**		See comment	1,180 (15 studies)	⊕⊕⊕⊖ moderate^1^	
	698 per 1,000	900 per 1,000 (858 to 949)				
	**Moderate**					
	727 per 1,000	938 per 1,000 (894 to 989)				
SCR		The mean new outcome in the intervention groups was 55.12 lower (63.42 to 46.82 lower)		1066 (13 studies)	⊕⊕⊕⊖ moderate^1^	
BUN		The mean bun in the intervention groups was 3.73 lower (4.08 to 3.37 lower)		1392 (17 studies)	⊕⊕⊕⊖ moderate^1^	
Upro		The mean upro in the intervention groups was 0.48 lower (0.53 to 0.43 lower)		1569 (19 studies)	⊕⊕⊕⊖ moderate^1^	
CCR		The mean ccr in the intervention groups was 9.48 higher (8.73 to 10.24 higher)		529 (6 studies)	⊕⊕⊕⊖ moderate^1^	
ArE		The mean new outcome in the intervention groups was 0.02 higher (0.02 to 0.02 higher)		173 (2 studies)	⊕⊕⊖⊖ low^1^	
Adverse reactions	**Study population**		OR 0.9 (0.48 to 1.69)	508 (6 studies)	⊕⊕⊕⊖ moderate^1^	
	87 per 1,000	79 per 1,000 (44 to 138)				
	**Moderate**					
	70 per 1,000	63 per 1,000 (35 to 113)				

^*^The basis for the assumed risk (e.g., the median control group risk across studies) is provided in footnotes. The corresponding risk (and its 95% confidence interval) is based on the assumed risk in the comparison group and the relative effect of the intervention (and its 95% CI). CI, Confidence interval; RR, Risk ratio; OR, Odds ratio; GRADE Working Group grades of evidence. High quality: Further research is very unlikely to change our confidence in the estimate of effect. Moderate quality: Further research is likely to have an important impact on our confidence in the estimate of effect and may change the estimate. Low quality: Further research is very likely to have an important impact on our confidence in the estimate of effect and is likely to change the estimate. Very low quality, We are very uncertain about the estimate.

^1^ No explanation was provided.

## 4. Discussion

### 4.1. Evaluation clinical efficacy and safety

CRF is a chronic progressive renal parenchymal injury disease with partial or complete loss of renal function due to various reasons such as chronic glomerulonephritis. CRF is characterized by slow progress and irreversibility, and manifested as facial and eyelid edema, nausea, vomiting, abdominal distension, fatigue, dysuria, proteinuria, hematuria, etc., (40–42). CRF will cause a series of complex damage to the kidney, thus inducing chronic systemic inflammation without significant clinical manifestation. This kind of inflammation is classified in immune inflammations, not equivalent to a systemic inflammatory response syndrome (SIRS) or a microbial infection (3). Alprostadil, a kind of prostaglandin E1 preparation, exerts its role in expanding blood vessels and reducing peripheral resistance. On this basis, vascular smooth muscle can be relaxed, and microcirculation can be improved, thus preventing glomerular thrombosis. That will achieve the goal of reducing proteinuria, increasing blood flow, and inhibiting inflammatory mediators. Renal function protection can be achieved through glomerular sclerosis (43, 44). However, alprostadil has certain adverse reactions, such as redness, pain, itching at the injection site, and even causes chest tightness and blood pressure drop. CRF is traditionally considered as blood stasis and water dampness based on the deficiency of kidney and spleen (43). Rhubarb, salvia miltiorrhiza, astragalus, and safflower in SKI have the functions of activating blood circulation and removing stasis, which can clear organs, remove dampness, invigorate qi and promote blood circulation. Modern pharmacological research have shown that Shenkang injection can improve renal tubular reabsorption, inhibit the proliferation of mesangial cells and renal interstitial fibrosis, promote the recovery of renal function, reduce renal tubular injury, and promote Ccr excretion (45). According to relevant studies, the combined treatment of SKI and alprostadil can achieve favorable efficacy in terms of CRF caused by diabetes and chronic kidney disease in elderly patients, with effectively reduced BUN and alleviated condition (46).

In our results showed that the overall response rate (ORR) of the treatment group was superior to the control group [RR = 0.20, 95% CI (0.16, 0.25), *P* < 0.00001]. Compared with the control group, the treatment group achieved favorable improvement in terms of the creatinine clearance rate (Ccr) [MD = 9.48, 95% CI (8.73, 10.24), *P* < 0.00001], serum creatinine (Scr) [MD = −55.12, 95% CI (−63.42, −46.82), *P* < 0.00001], quantitative urine protein (Upro) [MD = −0.48, 95% CI (−0.53, −0.43), *P* < 0.00001], and blood urea nitrogen (BUN) [MD = −3.73, 95% CI (−4.08, −3.3) 7, *P* < 0.00001]. There was no statistical difference in the incidence of adverse reactions in each group.

All in all, the combined treatment of SKI and alprostadil exhibited great advantage in CRF. Meanwhile, the results of Meta-analysis remained unchanged after removing some documents, indicating the stablity and reliablity of the results. Adverse reactions were specifically reported in only 6 papers, without serious adverse ones. There was no statistically significant difference between the two groups.

### 4.2. Limitaitons of the study

(1) Publication bias may exist in this study due to the small number and sample size, uneven course of treatment and Chinese language resources of the included literature; (2) All the studies were RCT, but most of them did not describe the specific random sequence generation method and the implementation scheme of allocation concealment, resulting in some limitations of the research results; (3) The great statistical heterogeneity of BUN indicators cannot be strictly eliminated according to the inclusion and exclusion criteria. (4) Adverse reactions were not reported in most studies. Great importance should be attached to relevant literature at home and abroad to improve the systematic evaluation, so as to more accurately draw conclusion on the efficacy of SKI combined with alprostadil in CRF treatment.

A total of 20 clinical RCTs were included in this study and analyzed through evidence-based medicine. The sound efficacy of SKI combined with alprostadil may shed new light on integrated traditional Chinese and western medicine in CRF treatment. However, there is still a long way to go in CRF treatment with large-scale and high-quality RCTs due to low-quality literature included in this study.

## 5. Conclusion

The current evidence indicates that therapy of SKI combined with Alprostadil for CHF positive efficacy and high safety. It is also simple, convenient. However, because of the limitations of the follow-up period and publication bias of the included trials, more rigorous clinical studies are necessary to further verify the long-term effects of it.

## Data availability statement

The original contributions presented in the study are included in the article/supplementary material, further inquiries can be directed to the corresponding author.

## Author contributions

TZ and FX designed the study, carried out statistical analysis, and revised/reviewed the manuscript. XQ and TZ carried out literature research. ML and PZ acquired data. TZ and PZ edited the manuscript. WL approved the final version of the manuscript. All authors contributed to the article and approved the submitted version.
